# The *Vave* campaign: impact evaluation of a cancer awareness raising multi-media campaign in Samoa

**DOI:** 10.1093/heapro/daac021

**Published:** 2022-03-26

**Authors:** Ernesta Sofija, Neil Harris, Beatriz Cuesta-Briand, Tim Spratling, Shelley Burich

**Affiliations:** 1 School of Medicine and Dentistry, Griffith University, Gold Coast Campus, Parklands Drive, Southport, QLD 4222, Australia; 2 Samoa Cancer Society, Apia, Samoa; 3 Rural Clinical School, The University of Western Australia, 35 Stirling Highway, Crawley, WA 6009, Australia

**Keywords:** Pacific Islands, community education, cancer prevention

## Abstract

Cancer is a leading cause of premature death and disability in Samoa. Recognizing the importance of symptom awareness and early detection, the Samoa Cancer Society (SCS) developed the ‘*Vave’* (quickly) campaign as the first multi-media cancer awareness campaign in Samoa. The campaign adopted a three-pronged community engagement approach including mass media; printed resources; and community outreach at culturally appropriate locations including churches, villages and schools. The campaign promoted three key messages: detect signs and symptoms quickly; quickly see a doctor; and quickly call SCS. To measure impact, data were collected using several methods around the outreach education sessions (pre- and post-surveys), campaign recall (survey) and *Vave*-related enquiries received by SCS. The findings revealed the campaign was effective in increasing awareness of cancer and importance of early detection demonstrated through community recall of campaign messages, increased enquiries to SCS and improved knowledge. However, it is of note that almost 30% of campaign recall respondents stated they were unsure or would not see a doctor if concerned about a sign of cancer. The reasons given being a lack of knowledge, lack of trust in hospitals and preference for traditional healing. This suggests more targeted culturally sensitive strategies are needed including partnering with traditional healers. Further, advocacy efforts are needed to address the structural barriers to cancer detection and treatment together with continuing education around causes and symptoms of cancer targeting the hard-to-reach communities in Samoa.

## BACKGROUND

Cancer is a leading cause of premature death and disability worldwide (Wild *et al*., 2020). Although cancer incidence is greater in high-income countries, the number of people affected by cancer is increasing in the World Health Organization’s (WHO) Western Pacific Region, including Samoa where cancer is a leading cause of death (Ministry of Health [MOH], 2015; Sarfati *et al.*, 2019). Samoa is a small island nation of ∼195 000 people spread across six islands with three-quarters of the population living in and around the capital of Apia on the island of Upolu (Samoa Bureau of Statistics [SBS], 2016). Several factors contribute to the growing cancer burden in the region including increased urbanization, lifestyle changes, inequitable access to health care, cultural barriers to early detection and treatment and an ageing population (Varghese *et al.*, 2014; Sarfati *et al.*, 2019).

There is an absence of recent reliable epidemiological data on cancer in Samoa (Dachs *et al.*, 2008; Moore *et al.*, 2010; WHO, 2014; Tervonen *et al.*, 2017) with cancer rates being estimated based on data from neighbouring countries such as American Samoa, Fiji or Vanuatu (Varghese *et al.*, 2014; Youlden *et al.*, 2014), or on previous epidemiological data for the period 1980–88 (Dachs *et al.*, 2008). It is estimated that in Samoa in 2012, there were 63 incident cases of cancer among men (age standardized rate of 92.5 per 100 000) and 74 incident cases among women (age standardized rate of 96.1 per 100 000), with an estimated 43 and 38 cancer deaths among men and women, respectively (64.4 per 100 000 men and 49.4 per 100 000 women) (Varghese *et al.*, 2014). Hospital surveillance data for the period 2005–09 identified gastrointestinal, breast cancer, lung cancer and prostate cancer as the most common types of cancer (Kulkarni and Dean, 2010).

Early detection and early intervention increase the likelihood of successful cancer treatment (Allgar and Neal, 2005). Data collected by the Samoa Cancer Society (SCS) suggest that many patients present late, and due to the absence of chemotherapy and radiation treatment services locally, treatment options for these patients are limited (Cuesta-Briand *et al.*, 2021a). Chemotherapy and radiation treatment are available through Samoa’s Overseas Treatment Scheme, which covers the cost of cancer treatment in New Zealand or India (SCS, 2018, Cancer patient registry, unpublished data). However, eligibility criteria include the determination of a high rate of treatment success, which is often associated with early diagnosis (SCS, 2018, Cancer patient registry, unpublished data). The resulting situation leaves those with late diagnosis with limited treatment options, often reduced to receiving palliative care at home (SCS, 2018, Cancer patient registry, unpublished data). This represents a significant economic and psychosocial burden on the families of cancer patients, who may be unable to provide adequate in-home care and support (MOH, 2015).

International evidence shows that the reasons for delayed presentations among cancer sufferers are complex and multifactorial, and include demographic, clinical, financial, psychosocial and cultural factors (Macleod *et al.*, 2009; Cuesta-Briand *et al.* 2021a). Research conducted with Samoan populations living in the USA showed that there is a belief that cancer is not a Samoan illness but, rather, an illness brought by Westerners (Hubbell *et al.*, 2005), and that cancer cannot be cured and means death (Ishida et al., 2001). Further, cultural and psychological factors such as modesty and fear, play a role in attitudes towards cancer screening hindering early detection (Ishida et al., 2001; Mishra *et al.*, 2007). In Samoa, unpublished research conducted by SCS showed low levels of health literacy in relation to cancer symptoms, risk factors and treatment among the general population, together with some confusion with other diseases and health messages (SCS, 2012, 2017, Market surveys, unpublished data). In addition, data from a WHO pilot study conducted with Samoan cancer patients found that many had relied on traditional medicine pre-diagnosis and that lack of trust in the healthcare system resulted in delays to seeking diagnosis and treatment (WHO, 2016, Insight into the experiences of patients seeking healthcare in Samoa, unpublished data). A more recent study with Samoan cancer patients corroborates these findings highlighting the importance of cultural barriers to early detection (Cuesta-Briand *et al.*, 2021a). Churches in Samoa play a central role in Samoan culture and village life making churches an appropriate place for health promotion (Holmgaard, 2019). Addressing low cancer awareness and cancer-related health literacy in the community at culturally appropriate locations, such as churches, schools and villages, has the potential to reduce barriers to early detection.

Mass media has been used to communicate health messages across a number of public health issues including alcohol consumption, tobacco use, physical activity, sexual health, cancer screening, road safety and immunization (Snyder et al, 2004; Wakefield *et al.*, 2010; Saleh *et al.*, 2012; Sugden *et al.*, 2017). Whilst the evidence on the effectiveness of mass media campaigns is mixed, research shows that these campaigns are most effective in leading to behavioural change when combined with clinical and community-based interventions (Snyder *et al.*, 2004; Wakefield *et al.*, 2010). In the Pacific region, multifaceted public health campaigns have been implemented targeting HIV/AIDS prevention (Sewak and Singh, 2017), the elimination of lymphatic filariasis (King *et al.*, 2011) and weight loss (Englberger et al, 1999; Baumann *et al.*, 2004), with encouraging results, indicating that the development and implementation of a media campaign may be beneficial for cancer diagnosis and treatment. As such, recognizing the importance of increasing symptom awareness to promote early detection, the SCS implemented the ‘*Vave’* (quickly) campaign. The *Vave* campaign was the first cancer symptom awareness multi-media campaign to be implemented in Samoa. This paper presents an impact evaluation of the campaign implemented between March 2017 and May 2018.

## THE *VAVE* CAMPAIGN

The *Vave* campaign was designed to raise community awareness of cancer signs and symptoms and the importance of acting quickly for early detection. The campaign promoted three key messages: (i) detect the signs and symptoms quickly; (ii) quickly see a doctor; and (iii) quickly call SCS. As the most common types of cancer in Samoa, gastrointestinal, breast cancer, lung cancer and prostate cancer (Kulkarni and Dean, 2010) were each targeted for a 3-month period. To maximize reach, especially among communities with limited access to news media, the campaign adopted a three-pronged community engagement approach including mass media and social media; printed resources; and community outreach. Community outreach sessions delivered at churches, villages and schools were included to reduce cultural barriers to participation.

*Mass media and social media* included television and radio advertisement, television and radio talk-back interviews, mobile text messaging, media releases and one billboard at a highly visible location in Apia. The campaign messaging, logo and image were added to SCS Facebook page with the page regularly updated with photos, videos and posts on *Vave* events and activities.

*Printed resources* included four brochures, each focussing on one of the four types of cancer targeted during the campaign, and available in Samoan and English. The brochures included information on risk factors, prevention, symptoms, diagnosis and treatment. Brochures were distributed at community education sessions and General Practice Clinics. In addition, A3 posters and pull-up banners were designed with the campaign logo, image and messaging. The posters were displayed at the hospital and distributed to GP clinics and other locations such as the Ministry of Health head office. The banners were displayed at the main Hospital and during the community education sessions.

*Community outreach* comprised face-to-face cancer awareness education sessions, targeting church groups, villages and schools in geographic areas with limited access to news media. The purpose of the community outreach education sessions was to increase cancer-related health literacy and address some cultural myths around cancer. In addition, the sessions provided information on cancer services available in Samoa, including the role of SCS, the overseas treatment scheme, and the provision of basic palliative care at home. A total of 31 sessions were delivered during the campaign, involving 2788 community members; this represents ∼1.4% of the Samoan population. The majority of the sessions (*n* = 19) were conducted on the island of Upolu, while the rest were conducted in Savai’i (*n* = 6), Manono (*n* = 4) and Apolima (*n* = 2). In addition to the community outreach education sessions, the *Vave* campaign was promoted with resources distributed at community events, such as: the official launch of World Breastfeeding Week in August 2017, The World Cancer Day community event hosted by the Ministry of Health in February 2017 and The Teuila Festival in September 2017.

## METHODS

To measure the short-term impact of the campaign, data were collected using several complementary methods around the outreach education sessions, campaign recall and *Vave-*related enquiries. The outreach education sessions were evaluated through brief pre- and post-surveys administered in Samoan. The surveys were distributed at 15 of 31 sessions. Participants were asked how strongly they agreed with eight statements (e.g. ‘Cancer only affects adults’) relating to the content from the sessions with responses recorded through a five-point Likert-scale response format with scores ranging from 1 (Strongly Disagree) to 5 (Strongly Agree). In total 210 respondents completed the surveys. Prior to analysis, data were screened with non-matched and largely incomplete surveys removed (*n* = 94) resulting in a final sample of 116. Completed matched surveys with some missing values were retained and missing values imputed with the median of nearby points (missing rate of 5.15%). Distribution was assessed as not normal using Shapiro–Wilk and Kolmogorov–Smirnov tests. Therefore, for comparison of pre- and post-results, the Wilcoxon Signed Rank test was conducted at the 95% significance level (α = 0.05) using the SPSS version 24.0.

To measure the campaign recall, a paper-based survey was administered in the weeks following the end of the campaign in May 2018. The survey instrument collected basic demographic information together with nine questions around community awareness and recall of the campaign’s main messages. Questions used dichotomous (yes/no) and Likert scale type response options. Two trained data collectors administered the survey to a convenience sample of 205 Samoan residents 18 years and older at various locations in the Apia town area. Descriptive statistics were used to analyse the campaign recall survey data. Finally, *Vave-*related enquiries received by SCS were monitored on an ongoing basis to understand whether the community members acted upon the key campaign messages—‘quickly call us’.

The ethics approval for this study was granted by Griffith University Human Research Ethics Committee (GU Ref. No.: 2020/736) and the use of previously collected de-identified data for research purposes was approved by the SCS.

## RESULTS

### Outreach education sessions

Overall, attitudes/knowledge of participants improved significantly after the outreach education sessions (*p* = 0.038). Table 1 presents results from pre- and post-questionnaires. The results show significant improvement in knowledge/attitudes for Q3 *If cancer is found early, it can be treated* (*p* = 0.004), Q7 *Only traditional medicine can cure cancer* (*p* = 0.039) and Q8 *If I had a symptom of cancer, I would know what to do* (*p* = 0.01).

**Table 1:** Differences between pre-test and post-test scores for cancer awareness education sessions

PreT/PosT items	Measures of central tendency				Measures of difference	
	T1 Mean (±SD)	T2 Mean (±SD)	T1 Median	T2 Median	*Z*	*p* value
1. Cancer only affects adults	2.573 (±1.31)	2.516 (±1.44)	2	2	−0.340	0.734
2. Cancer only affects people who drink or smoke	2.526 (±1.17)	2.487 (±1.24)	2	2	−0.518	0.605
3. If cancer is found early, it can be treated	3.853 (±1.28)	4.633 (±1.03)	4	4.5	−2.862	0.004^*^
4. Cancer can affect anybody	3.634 (±1.39)	3.647 (±1.38)	4	4	−0.099	0.921
5. Losing weight can be a symptom of cancer	3.034 (±1.18)	3.263 (±1.22)	3	3	−1.888	0.059
6. Smoking is a risk factor for cancer	3.74 (±1.35)	3.67 (±1.33)	4	4	−0.530	0.596
7. Only traditional medicine can cure cancer	2.177 (±0.99)	1.945 (±1.20)	2	2	−2.069	0.039^*^
8. If I had a symptom of cancer, I would know what to do	3.366 (±1.13)	3.772 (±1.34)	3	4	−2.576	0.010^*^

* *p *<* *0.05.

### *Vave*-related enquiries

**Fig. 1:**
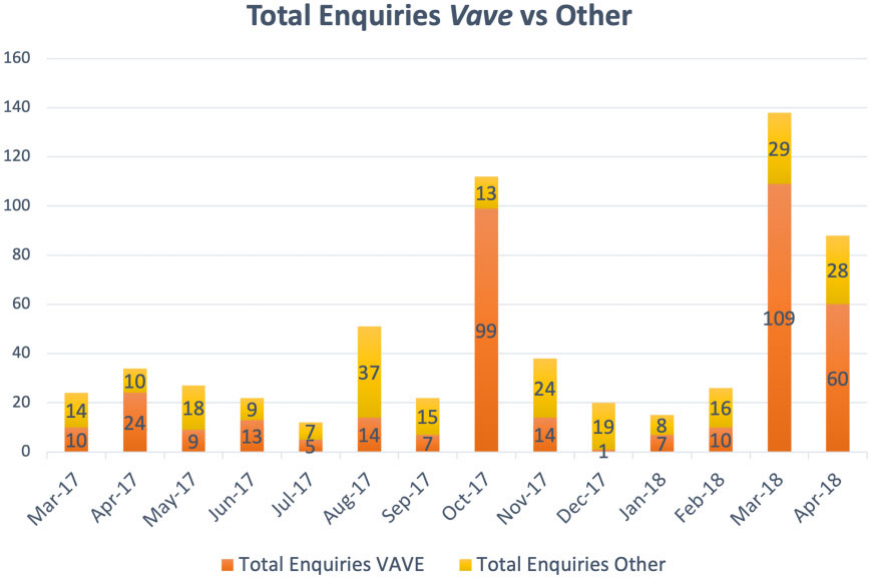
Number of *Vave*-related enquiries, other enquiries and total number of enquiries (March 2017–April 2018).

In the 4-month leading up to the campaign (November 2016 to February 2017), SCS received a total of 70 enquiries, this is an average of 18 enquiries a month. Aligned with the intervention objective of ‘quickly call us’, the number of enquiries received at SCS experienced a marked increase during the period March 2017 to April 2018 (Figure 1). In the early stages of the campaign (March 2017), *Vave*-related enquiries represented 42% of the total number of enquiries received by SCS (10 of 24). This proportion increased to 60% in April (24 of 34), and peaked during October (coinciding with *Pinktober—breast cancer awareness month*) to represent 88% of enquiries (99 of 112) and again in March 2018 (following the prostate cancer campaign), representing 79% of enquiries (109 of 138). Enquiries related primarily to signs and symptoms of cancer, where to go for treatment, information about SCS and how the society could help. Throughout the campaign a total of 629 enquiries were received. This is an average of 45 enquiries a month and represents an increase of 27 enquiries a month, attributable to the *Vave* campaign.

### Campaign recall

Samoan residents (*n* = 205) were surveyed about their recall of the campaign, its messaging and the types of cancer targeted (see Table 2 for further detail). Two-thirds of respondents (68.8%, *n* = 141) remembered the campaign. Of those respondents who remembered the campaign the majority (80.8%, *n* = 114) heard about it on the TV with only three (2.1%) respondents reading about it in the newspaper.

**Table 2:** Campaign recall survey results (*n* = 205)

Question	Respondents (*n*)	Respondents (%)
Remember campaign (*n* = 205)		
Yes	141	68.8
No	64	31.2
If yes, how did they hear about it (*n* = 141)		
TV	114	80.8
Radio	65	46.1
SMS	18	12.8
Social media	17	12.1
Newspaper	3	2.1
Other (through work, word of mouth, outreach session or visiting SCS)	6	4.3
Remember messages (*n* = 141)		
Yes	127	90.1
No	14	9.9
If yes, what messages (*n* = 127)		
Early detection	45	35.4
See a doctor	101	79.5
Call us	43	33.9
Remember types of cancer (*n* = 141)		
Yes	134	95.0
No	7	5.0
If yes, what types of cancer (*n* = 134)		
Stomach cancer	1	0.7
Lung cancer	19	14.2
Breast cancer	64	47.8
Prostate cancer	113	84.3
Would you go to the doctor if you were concerned about any signs and symptoms of cancer? (*n* = 205)		
Yes	149	72.7
Unsure	31	15.1
No	25	12.2

As shown in Table 2, most respondents (90.1%, *n* = 127) who recalled the campaign, remembered one or more of the campaign messages with the highest recall being the message ‘Quickly see a Dr’ (*Vave va’ai se fomai*) (79.5%; *n* = 101). Of those who remembered the campaign, a total of 134 participants (95.0%) remembered one or more of the cancer types targeted by the campaign.

The campaign was effective in increasing awareness of the importance of early detection and seeing a doctor. A total of 113 participants (80.1%) agreed that the campaign had increased their knowledge of the importance of (i) early detection and (ii) seeing a doctor, while 23 (16.3%) were unsure and only five (3.6%) disagreed. Survey results suggest the campaign was, by comparison, less effective in increasing knowledge about the signs and symptoms of cancer, although more than half of those who remembered the campaign agreed that they now knew more about signs and symptoms (58.1%, *n* = 82), while 40 (28.4%) were unsure and 19 (13.5%) disagreed.

Finally, whether respondents remembered the campaign or not, they were asked whether they would see a doctor if they were concerned about a sign or symptom of cancer with almost three quarters indicating that they would (72.7%, *n* = 149) and 56 respondents stated they were unsure or would not see a doctor (15.1% and 12.2%, respectively). The most common reasons for being unsure or not seeing a doctor included: lack of knowledge about the signs and symptoms of cancer or about when to see a doctor (*n* = 16); lack of trust in doctors and hospitals (*n* = 5); and preference for traditional Samoan healing (taulasea) (*n* = 5). Other stated reasons included: cancer having no cure (*n* = 2), fear of having an operation (*n* = 1) and one respondent stated that ‘no Dr is smarter than God’.

## DISCUSSION

The *Vave* campaign was the first cancer symptom awareness multi-media campaign to be implemented in Samoa. The campaign was effective in increasing community awareness of cancer and the importance of early detection and seeking help. This is particularly significant as at the patient level, reasons for delays in cancer diagnosis include poor awareness of symptoms, negative beliefs about cancer outcomes and poor awareness of cancer risks (Croager *et al.*, 2018). The *Vave* campaign was not, strictly speaking, a social marketing campaign (Andreasen, 2002). However, the campaign adopted marketing principles, characteristic of social marketing campaigns, including a clear call for action: detect the symptoms early, then see a doctor or call SCS. Thus, *Vave* was behaviour oriented, that is, it focussed on changing people’s behaviour, not just informing or educating, and the behavioural objectives were specific and measurable (Sewak and Singh, 2017).

The outreach community education component of *Vave* was effective in increasing community awareness of cancer. The community education pre- and post-questionnaire results revealed an increased understanding of cancer detection and treatment among participants. Interestingly, community understanding of risk factors and symptoms did not show significant improvement. The improved cancer awareness achieved through the community outreach education sessions resonates with the acknowledged importance of taking a grassroots approach to reaching underserved and/or geographically remote communities (Northington *et al.*, 2011). The lack of improvement reported around understanding the risk factors and cancer symptoms is consistent with previous research that has suggested cultural norms and beliefs that cancer is a western illness are acting as barriers to raising community awareness (Cuesta-Briand et al., 2021a,b). This suggests the need to draw in community partners such as traditional healers, chiefs and church leaders to support multiple strategies that address these misconceptions to increase depth of community knowledge.

The findings from the campaign recall survey demonstrate that the majority of respondents remembered the *Vave* campaign and its key messages. The *Vave* campaign used short and sharp, catchy messages that were repeated throughout the campaign, which may have contributed to a relatively high level of recall. Literature suggests that message repetition contributes to the effectiveness of multimedia campaigns (Englberger *et al.*, 1999; Shi and Smith, 2016). TV and radio advertisements and programmes were found to be the most commonly recalled medium for campaign messaging. This finding is consistent with other multimedia campaigns implemented in the Pacific region (Englberger *et al.*, 1999) and more recent studies in other locations (e.g. Malaysia/Scotland/England: Eadie *et al.*, 2009; Saleh *et al.* 2012; Martin *et al.*, 2018). It is somewhat surprising that SMS and social media were far less commonly recalled mediums, however, this may reflect the cost of telephone and Internet usage in the country (The World Bank, 2013). The lack of newspapers as a medium reflects the global decline of print media.

The campaign was effective in encouraging community members to take action as seen in an increased number of enquiries regarding cancer screening. The data shows that enquiries to SCS during the campaign increased dramatically from 18 to 45 per month with enquiries peaking during breast and prostate cancer awareness months. This indicates that the campaign had increased awareness in community and the message to take action (‘*quickly call us*’) was effective. This is a promising finding as research into cancer awareness campaigns often report increased awareness and intentions to adjust behaviour but not actual change in behaviour (Dixon et al., 2015; Martin *et al.*, 2018). To further understand the intermediate impacts of multi-media cancer awareness campaigns in Samoa, future studies could investigate the changed patterns in cancer-related check-ups and screening requests attributable to these campaigns. To enable this, SCS could collaborate with the Ministry of Health to access screening data both pre- and post- delivery of the *Vave* campaign and engage clinicians to record reasons for new patients seeking screening.

Ultimately, the campaign’s call to action relies on the availability of adequate healthcare services. Access to healthcare services in Samoa is hindered by geographic barriers, with most screening services and GP surgeries located on the main island, and financial barriers relating to the cost of implementing screening and treatment services as well as out-of-pocket costs to community members (SCS, 2018, Cancer patient registry, unpublished data). Other structural barriers relate to healthcare system issues such as lack of clear referral pathways, limited screening services and gaps in clinical expertise among some health professionals (SCS, 2018, Cancer patient registry, unpublished data; Sarfati *et al.*, 2019). Addressing individual-level barriers to early detection is necessary, however, structural barriers to early detection must be addressed and cancer services strengthened if better cancer outcomes for patients in Samoa are to be achieved. Previous research suggests that mass-media campaigns around cancer awareness can increase related policy support at the population level (Martin *et al.*, 2018). Raising awareness around cancer in Samoa may lead to increased discussions around the mix of health services available together with increased emphasis on cancer prevention. As Sarfati *et al.* (2019) posited, cancer control in Samoa will require regional partnerships, given the size of the country and economic situation whereby local extensive cancer services may not be feasible.

There were several limitations with the study. First, implementation of the community education pre- and post-questionnaires only commenced in September 2017, meaning reduced participation of this component of evaluation. Second, completion of a survey to collect quantitative data is not a routine happening in Samoa. As a result, the five-point Likert scale design of pre- and post-surveys proved unfamiliar and therefore difficult for some participants to complete. However, to overcome this issue, researchers were available to provide an explanation of the response options and how to complete the survey. In the future, it would be useful to design and trial a survey tool that replaces numbers usually used on Likert scales with more visually appealing response options. Such a strategy may help make programme evaluation tools more accessible to communities who may be less experienced in completing standard surveys. While the surveys linked with the community outreach sessions involved communities of 15 villages across the islands, the recall survey was only administered in and around the capital city of Apia. To address this limitation future research should use a more structured sampling strategy involving data collection at multiple sites and islands to recruit participants and thereby reach a broader cross-section of the community. Finally, this evaluation focussed on short-term impacts. Future studies should aim to understand intermediate and long-term impacts of such campaigns in Samoa.

## CONCLUSION

The *Vave* campaign was the first cancer symptom awareness multi-media campaign implemented in Samoa. Overall, the campaign was successful in increasing community awareness of cancer and the importance of early detection. Advocacy efforts will be needed to address some of the structural barriers to cancer early detection, as well as continuing education targeting the hard-to-reach communities in Samoa.

## ETHICAL APPROVAL

Ethical clearance for this research was obtained through Griffith University’s Human Research Ethics Committee (Ref. No.: 2020/736).
